# The potential of micro- and nanoplastics to exacerbate the health impacts and global burden of non-communicable diseases

**DOI:** 10.1016/j.xcrm.2024.101581

**Published:** 2024-05-22

**Authors:** Stefan Krause, Valerie Ouellet, Deonie Allen, Steven Allen, Kerry Moss, Holly A. Nel, Semira Manaseki-Holland, Iseult Lynch

**Affiliations:** 1 School of Geography, Earth and Environmental Sciences, University of Birmingham, Edgbaston, Birmingham B15 2TT, UK; 2 Institute for Global Innovation, University of Birmingham, Edgbaston, Birmingham B15 2TT, UK; 3 Birmingham Institute for Sustainability and Climate Action (BISCA), University of Birmingham, Edgbaston, Birmingham B15 2TT, UK; 4 Ecologie des Hydrosystèmes Naturels et Anthropisés (LEHNA), Université Claude Bernard Lyon 1, Lyon, CNRS, ENTPE, UMR5023, 69622 Villeurbanne, France; 5 WESP - Centre for Water, Environment, Sustainability & Public Health, Department of Civil and Environmental Engineering, University of Strathclyde, Glasgow G1 1XQ, UK; 6 Institute of Applied Health Research, University of Birmingham, Edgbaston, Birmingham B15 2TT, UK

**Keywords:** microplastic, additives, exposure, particle, inflammatory, inflammation, global health

## Abstract

Non-communicable diseases (NCD) constitute one of the highest burdens of disease globally and are associated with inflammatory responses in target organs. There is increasing evidence of significant human exposure to micro- and nanoplastics (MnPs). This review of environmental MnP exposure and health impacts indicates that MnP particles, directly and indirectly through their leachates, may exacerbate inflammation. Meanwhile, persistent inflammation associated with NCDs in gastrointestinal and respiratory systems potentially increases MnP uptake, thus influencing MnP access to distal organs. Consequently, a future increase in MnP exposure potentially augments the risk and severity of NCDs. There is a critical need for an integrated one-health approach to human health and environmental research for assessing the drivers of human MnP exposure and their bidirectional links with NCDs. Assessing these risks requires interdisciplinary efforts to identify and link drivers of environmental MnP exposure and organismal uptake to studies of impacted disease mechanisms and health outcomes.

## Graphical abstract

**Figure:**
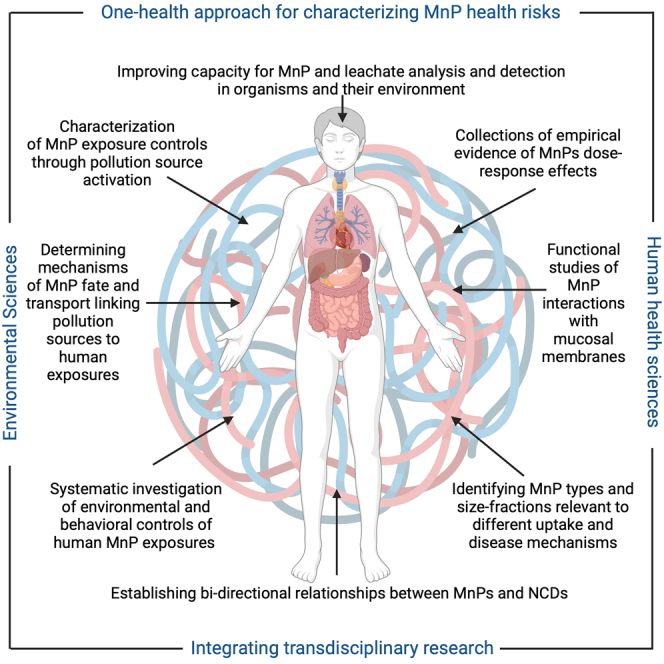

## Teaser

Krause et al. integrate existing knowledge of the impacts of micro- and nanoplastics on non-communicable diseases, evidencing that these emerging pollutants, together with their leachates, increase disease risk and severity. They advocate that the addressment of public health risks requires identifying the drivers of human exposure and pollution uptake mechanisms.

## Introduction: Risks of environmental MnP exposure and uptake

The incidence of non-communicable diseases (NCDs) is increasing globally.^1^ The four main types of NCDs (i.e., cardiovascular diseases such as heart attacks and stroke, cancers, diabetes, and chronic lung disease such as chronic obstructive pulmonary disease [COPD] and asthma) are collectively responsible for ∼71% of all global deaths annually,^1^^,^^2^ with a predicted economic impact of >$30 trillion over the next two decades.^3^ The global NCD burden, which quantifies health losses through both disability and mortality from NCDs and associated risks and costs to the health system, is known to be amplified by environmental pollution, compounding public health consequences.^2^^,^^4^

Global trends of environmental pollution show that micro- (≤5 mm) and nanoplastic (MnP; ≤1 μm) particles are now ubiquitous and found throughout the environment.^5^ Despite the growing evidence of the widespread environmental prevalence of MnPs, the health risks associated with MnP exposure are still uncertain.^6^^,^^7^ MnP particles have been detected in lungs, blood, breast milk, placenta, and stool samples^8^^,^^9^^,^^10^^,^^11^ (Table 1), confirming that MnP particles from the environment enter the human body.^12^ However, a current lack of synthesis of the mechanistic understanding of direct and indirect impacts of MnPs on human health as well as uncertainties arising from a lack of standardized extraction and analysis protocols, including assessment of cross-contamination, often prevent determining the actual health risks associated with this exposure.

**Table 1:** Types of plastic detected that passed the human body’s biological barriers or were excreted

Type of MnPs	Placenta	Meconium	Breast milk	Blood	Feces
Polyamide	✓	✓	✓	✓	✓
Polyurethane	✓	✓	✓	✓	✓
Polyethylene	✓	✓	✓	✓	✓
Polyethylene terephthalate	✓	✓	✓	✓	✓
Polypropylene	✓	✓	✓	✓	✓
Polyvinyl chloride	✓	✓	✓	✓	✓
Polyoxymethylene	✓	✓	✓	–	✓
Ethylene vinyl acetate copolymer	✓	✓	✓	–	✓
Polytetrafluoroethylene	✓	✓	✓	–	✓
Chlorinated polyethylene	✓	✓	✓	–	✓
Polybutadiene	✓	✓	✓	–	✓
Polycarbonate	✓	–	–	✓	–
Polystyrene	✓	–	✓	✓	✓
Polymethyl methacrylate	✓	✓	✓	✓	✓
Polylactic acid	✓	✓	✓	–	✓
Polysulfones	✓	✓	✓	–	✓
Nitrocellulose	–	–	✓	–	–
Size detected	detected: 5–10 μm/50–240 nm	>50 μm	2–50 μm	≥700 nm	infant: 20–50 μmadult: 50–500 μm
Reference	^13^^,^^14^^,^^15^	^13^^,^^15^^,^^16^	^13^^,^^17^	^7^^,^^10^^,^^18^^,^^19^	^11^^,^^13^^,^^19^^,^^20^^,^^21^

“✓” indicates the plastic type has been detected, whereas cells with the dash (–) indicate that no evidence of the presence of the MnP type was found in the corresponding medium.

With the current trend in plastic pollution estimating that by 2050, more than 12,000 metric tons of plastic waste will have accumulated in the environment or in landfills,^22^ we need to accelerate the quantification of human health risk associated with environmental MnP exposures. With this review, we want to draw attention to the potential reciprocal interactions between MnPs and NCDs. Based on the existing evidence, we hypothesize that the physical nature of MnPs and their chemical leachates impact the prevalence and severity of numerous NCDs by creating an internalized particle burden, potentially overwhelming antioxidant responses, and exacerbating existing low-level inflammatory responses in proximal and distal organs. Meanwhile, existing NCDs may enhance the uptake of MnPs, impacting individuals with pre-existing gastrointestinal (GI) or respiratory conditions through “leaky” epithelial barriers.^23^^,^^24^

**Figure 1:**
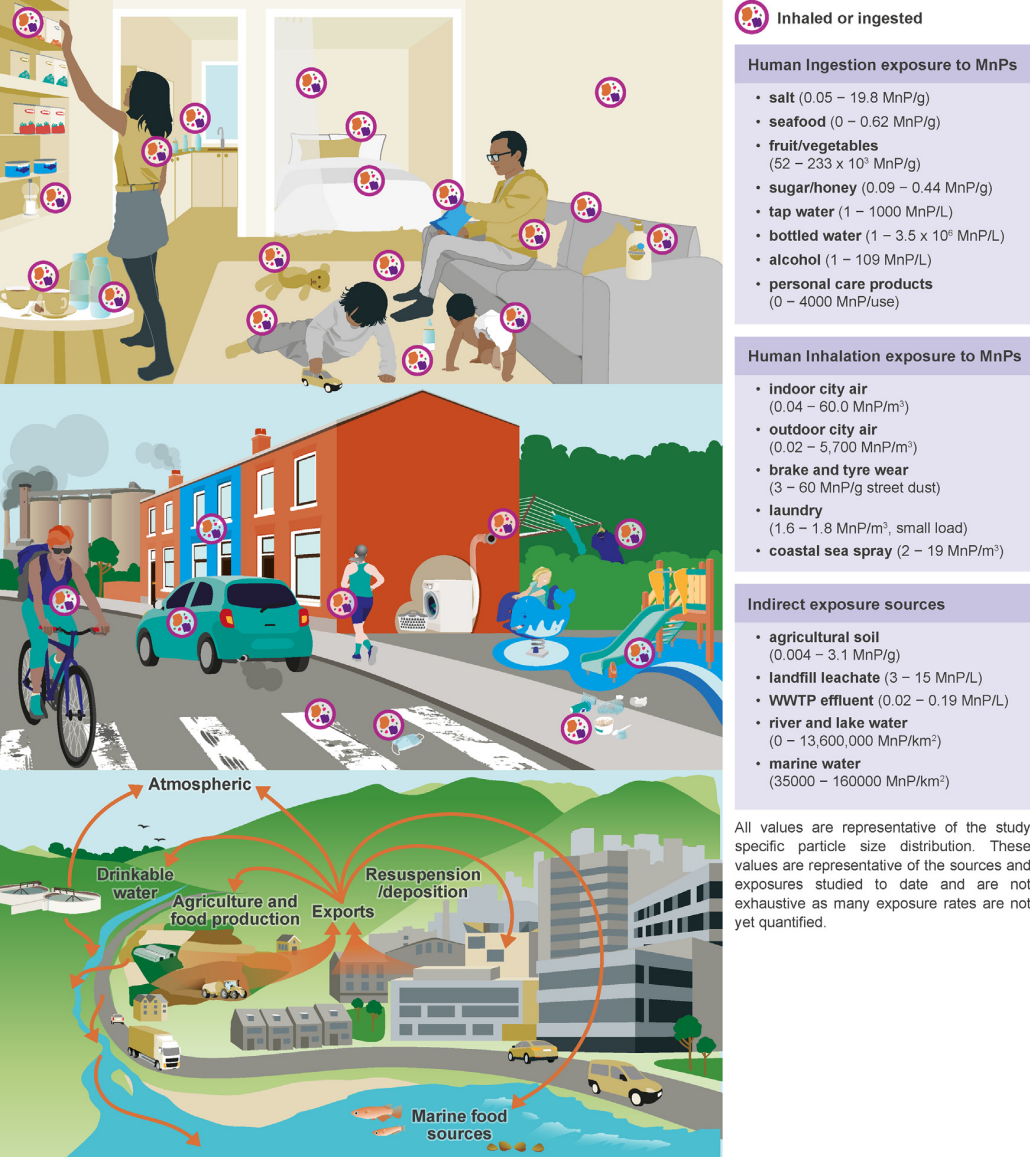
Environmental exposure routes, transport, and sources of MnPs Environmental exposure routes and sources of MnPs in indoor (top) and outdoor environments (middle). Human exposure rates are determined by the environmental fate and transport of MnPs that control the connectivity between spatially and temporally dynamic environmental pollution sources and human exposures (bottom). Together, these dynamic exposure controls determine the combined uptake of MnPs and their additives that may influence the risk and/or severity of NCDs. The text boxes provide some example exposure ranges associated with different MnP sources.

We highlight initial evidence that suggests that ingestion of MnPs can be linked to the same inflammatory and oxidative stress pathways associated with GI NCDs.^25^^,^^26^^,^^27^ Similarly, inhalation of MnPs can trigger inflammatory responses that resemble those associated with combustion-derived particulate matter (PM_2.5_).^28^^,^^29^ It is generally acknowledged that quantitative assessment of MnP exposure compared to other contaminants is limited, and researchers are only starting to identify MnP polymers and quantity numbers of MnPs in the human body.^10^^,^^13^,^14^,^17^ The widespread environmental prevalence of MnPs and their additives, their multiple exposure routes (Figure 1), and their various uptake mechanisms in humans, starting even before birth, highlight the human health relevance of any association between MnPs and NCDs explored in this review.

## Human exposure to MnPs

Although the quantification and identification of MnPs in the environment are still fragmented, several studies suggest that MnP concentrations in the environment have increased since the 1950s.^30^^,^^31^^,^^32^^,^^33^ Unsurprisingly, these patterns mirror the increase in global plastic production, use, and disposal in society.^22^ Plastics have become integral to daily life and activities (Figure 1), leading to widespread exposure and potential uptake routes of MnPs.^22^ This increases potential human health risks as, similar to other pollutants such as soot, vehicle-related carbon, asbestos, lead, and arsenic, MnP toxicity is related to exposure and dosage.^28^^,^^34^^,^^35^^,^^36^^,^^37^

Humans are exposed to MnPs in outdoor air^38^^,^^39^ and indoor environments,^40^^,^^41^ through food and food production processes, and via water/beverage consumption, among a multitude of other sources including cosmetics and human care products (Figure 1).^42^^,^^43^ Direct sources can include MnPs contained in food or beverages (e.g., fish, salt, beer, and plastic bottled beverages)^44^^,^^45^^,^^46^^,^^47^ and inhalation of MPs released by local emissions (e.g., MnPs released from plastic clothing, plastic fabric bedding during sleep, plastic carpet or furniture, MnPs released during sitting or walking).^9^^,^^48^^,^^49^^,^^50^ Indirect sources can include fertilizer, soil, atmospheric deposition or irrigation, MnP uptake into food crops or produce, contamination of ingestible products by MnP-rich soil or sediment (external transfer of MnPs),^27^^,^^51^ and inhalation of atmospheric MnPs from distal and diffuse sources (e.g., agricultural atmospheric MnPs transported to urban environments).^52^^,^^53^^,^^54^^,^^55^

**Figure 2:**
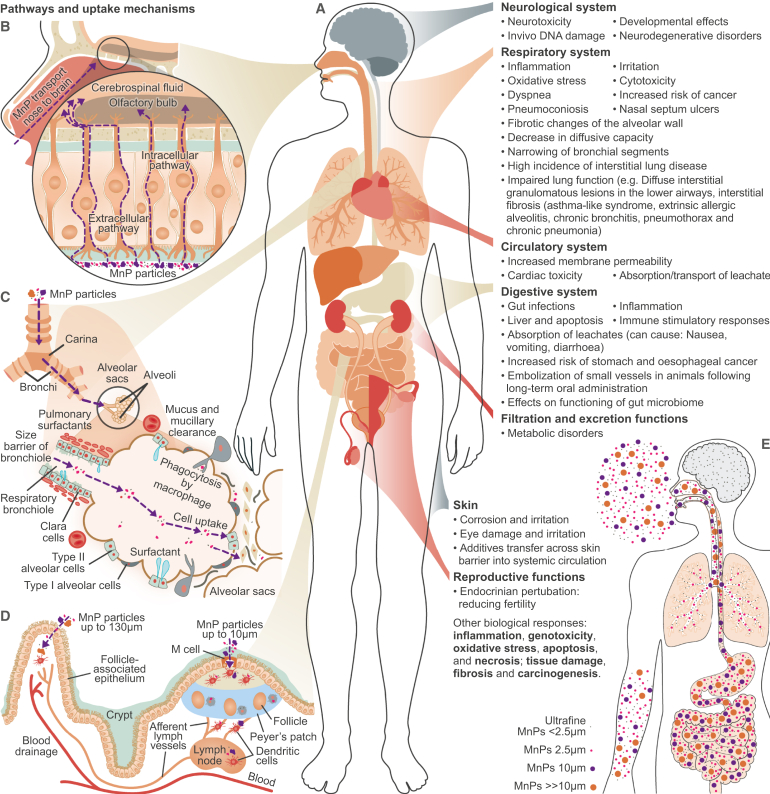
Hypothesized uptake mechanisms of MnPs through human body (A–D) (A) Hypothesized uptake mechanisms of MnPs through human biological barriers, including via (B) the olfactory bulb, (C) the lung-air barrier, and (D) the gastrointestinal tract, indicating also the systems and organs directly affected by MnPs and the associated MnP impacts and suspected adverse health outcomes including NCDs. The suspected particle-size fractionation caused by differences in the uptake mechanisms (A–D) is highlighted in (E), with larger particles being ingested (up to 130 μm) rather than inhaled (≤2.5 μm) and only the smallest (nanoscale) particles being able to penetrate the blood-brain barrier. MnP internalized by routes (C) and (D) reach the wider circulatory system and from there can reach all organs.

Given the complexity of different exposure routes, there is significant uncertainty about the relevance of different MnP uptake mechanisms. While direct skin exposure, for instance, can be high, it is generally assumed to result in lower uptake than other routes such as inhalation or ingestion, with evidence from the nanomaterials field indicating very little particle penetration through the skin, with even hair follicles having tight barriers preventing particles crossing into cells.^56^ A summary of potential uptake mechanisms of MnPs through human biological barriers (Figure 2) including emerging yet fragmented evidence for different routes is detailed in the next subsections.

### MnP uptake by inhalation

The inhalation of airborne MnPs has been confirmed by Jenner et al.,^9^ who revealed MnP uptake in the study of a small cohort of 11 patients, with polypropylene and polyethylene terephthalate fibers representing the most abundant MnP particles. While the full complexity of the disease mechanism after lung deposition remains to be explored, several studies indicate the potential of MnPs to contribute to cytotoxicity (Figure 2),^29^^,^^57^ with preliminary results suggesting that inflammation, oxidative stress, and physical cell damage can be cellular responses to MnP exposure.^58^^,^^59^^,^^60^ Yang et al.^61^ also highlighted that mice exposed to airborne MnPs expressed systemic inflammation and complete insulin resistance, featuring excessive drinking and eating, weight loss, elevated blood glucose, and decreased triglyceride levels; similar impacts may be found in humans.

Human exposure to airborne MnPs varies significantly depending on location and environment. Data on outdoor atmospheric MnP published to date suggest that atmospheric exposure concentrations in cities range from <20 (Paris, France, and Bushehr, Iran) to >100 MnP/m^3^ (Surabaya, Indonesia; Sakarya Province, Turkey; and Tianjin, Shanghai, Nanjing, and Hangzhou, China), with the highest reported exposure to date of 5,700 MnP/m^3^ (Beijing, China),^39^ even though limits of detection in methods applied for MnP identification and sampling and extraction protocols vary. Atmospheric MnP concentrations are influenced by the distance from the respective emission source, potential long-range transport mechanisms, and microclimatic drivers affecting dispersal at the deposition zone.^62^ For example, traffic can have a major influence, forming “pollution corridors” throughout a city,^63^^,^^64^^,^^65^ with profound impacts on individual exposure risks that even vary over time. Airborne MnP concentrations in rural (non-agricultural) to remote areas have been found to reach up to ∼20 MnP/m^3^.^39^ Airborne MnP exposure is suggested to be significantly greater in urban areas than it is in remote and rural regions, suggesting a higher risk of MnP inhalation or ingestion through particle deposition onto food and beverages.^55^^,^^66^

Several studies revealed indoor air in tested environments to contain >50 times higher MnP concentrations than were encountered in outdoor environments.^67^^,^^68^^,^^69^ While fewer studies of indoor air MnPs exist, they found indoor exposures ranging between 22 and 24,000 MnP/m^2^/day from sources such as carpets, wallpaper, furnishings, and clothing.^49^^,^^50^^,^^70^ Indoor atmospheric fallout studies identified the highest deposition rates in domestic housing, with small (5–250 μm in size) fibrous particles being the most abundant (90%) and MnPs (35–1,000 μm) constituting 2% to 8% of the remaining fallout deposition.^69^^,^^71^ The same studies also showed that polyethylene terephthalate, polyamide, polystyrene, polyvinyl chloride, and polypropylene were the most common polymer types of MnPs found in indoor air.^69^^,^^71^ Considering average inhalation rates and time spend indoors, the uptake of indoor MnP fibers has been estimated to be 11–44 MnP/kg body weight/day.^72^ Domestic housing has been shown to have elevated atmospheric MnP concentrations relative to office spaces, indicating that individual exposures to MnPs could be highest at home.^69^ Adult city inhabitants are estimated to inhale between 48,000 and 22,000,000 MnP/year^27^^,^^39^^,^^73^ and potentially orders of magnitude greater when living in highly polluted city locations. It is noted that these estimations of MnP uptake were calculated using literature-derived atmospheric MnP concentrations (averaged for city/urban environments) and respective country-specific guidelines for expected adult daily inhalation volume (e.g., 3.4–19.3 m^3^/day).^50^^,^^72^^,^^73^

### Ingestion of MnP

MnPs have been quantified in human fecal matter of adults and infants (Table 1), indicating that a proportion of ingested MnPs enter the GI system (∼28 MnP/g colon tissue, particles 0.8–1.6 mm^74^ and up to 36 MnP/g^20^^,^^75^). MnP concentrations in infant fecal matter were found to be of an order of magnitude higher than in adults.^13^ A possible explanation for higher MnP content in infant fecal matter is that plastic is commonly used in infant food preparation, presentation, and storage, which may result in higher specific ingestion in infants,^75^^,^^76^ or it may also be related to behavioral aspects (e.g., putting objects in their mouth) and their closer vicinity to and contact with indoor furniture. The use of linear extrapolation of exposure and uptake from adult values to infants (or simplistic body weight estimation) may therefore be inappropriate when estimating health risks.

Despite existing evidence for MnP occurrence in food, mechanistic understanding of the ratio of exposure to uptake or exposure to retention (transfer past the GI system into other organs or systems) remains unclear, limiting the current ability to link exposure information to expected uptake (Figure 2). Early studies of MnP exposure via food have focused predominantly on marine food sources (mussels, fish, and other seafood),^77^ which is certainly related to the higher awareness of MnPs in marine systems (Figure 1). However, more recent studies have also established potential exposure and uptake through salad and other agricultural produce.^78^^,^^79^ MnPs have been quantified in several forms of salt, honey, beverages, bottled and tap water, and packaged meat^42^^,^^44^^,^^75^^,^^80^ (Figure 1), and MnP ingestion has been linked to a variety of baby products (e.g., bottles, silicon teats).^76^^,^^81^ MnP exposure via drinking water is of great enough concern for the state of California (USA) to monitor MnP concentrations for a better understanding of exposures through drinking water with the aim to determine thresholds for MnP concentration standards to be brought into effect upon the existence of sufficient evidence (California’s Safe Drinking Water Act).^82^ Based on existing exposure values, adult MnP ingestion is estimated to range from 46,000 to 1,300,000 particles/year (equivalent to approximately 287 g/year, depending on diet),^27^^,^^73^^,^^83^ which is comparable to the estimated adult inhalation of MnPs mentioned above. (The MnP/m^3^ concentration is highly sensitive to the limit of quantification. There is a power-law distribution of MnP particles, with orders of magnitude greater quantities of particles as the particle size decreases.^84^ The relative limit of quantification has to be taken into consideration when comparing published findings.) While MnP ingestion occurs through the intake of different foods (table in Figure 1), actual individual MnP exposure may vary based on differences in the growing of produce and the specific harvesting, processing, and packaging mechanisms. Recent studies evidenced the preparation of food (atmospheric deposition during processing or cooking)^85^ and packaging^80^ as additional pathways of MnP exposure through food. Quite concerningly, there is growing evidence of exposure to MnPs during early life, with a significant number of MnPs found in formula milk^13^ and human breast milk (Table 1).^17^

### MnP material properties influencing human health risks

Human uptake of MnPs is impacted by particle properties such as size, shape, surface conditions, biomolecular-corona, i.e., the biological molecules that absorb to its surface, therefore changing the particle properties and giving it a biological identity,^86^ and environmental concentrations.^57^^,^^87^^,^^88^ However, substantial knowledge gaps exist about the retention and egestion rates of MnPs in the human body, with little mechanistic understanding of how material properties and shape, which are known to affect decomposition and degradation of MnPs, will influence their fate in organisms.^89^^,^^90^ From a human exposure and health perspective, the upper size spectrum of MnPs is not especially relevant,^91^ since larger MP particles would generally not be bioaccessible and therefore would be excluded from entering the body by the various biological barriers (Figure 2, lung-air barrier, gut barrier, olfactory system, and others). For comprehensive exposure risk assessments, it will be essential to improve the understanding of the sizes of particles passing through cell membranes, endothelial barriers, the blood-brain barrier, and placental barriers. Such information could support the proposition of a more mechanistically relevant MnP size classification according to groups related to human health and based on the particles’ ability to enter organisms through specific uptake mechanisms (Table 1).

MnPs >20 μm have been identified in human blood (1.6 μg/mL) and in human stool samples, evidencing MnP uptake and internal transportation (Table 1).^7^^,^^10^^,^^11^^,^^20^^,^^92^ This means that particles are both ingested and able to pass through the GI system. Smaller particles are even more likely to pass through the GI system but are usually below the detection limits of commonly applied analytical methods. The fact that particles are known to pass through the GI tract also suggests that there could be opportunities for them to be adsorbed and/or leach chemicals during their transit, as evidenced by the detection of MnPs in blood,^10^^,^^18^ placenta,^14^^,^^93^^,^^94^ and breast milk (Table 1).^13^^,^^17^ However, given the small number of studies on specific MnP intake, it is difficult to evaluate how common and representative the transport and potential uptake of MnPs during the ingestion and GI transport process are. For instance, polystyrene particles of 1 and 4 μm have been found to be effectively taken up by a Caco-2 monoculture and Caco-2 co-culture models with microfold cells (Peyer’s patches), while very low numbers of 10 μm particles were internalized in cell barrier models.^95^

The assessment of hazards and risks arising from MnP exposure is hampered by the limitations of currently available analytical methods deployed for detecting, identifying, and quantifying the presence of MnPs in biological tissue to establish exposure-effect responses (dose-response functions). Importantly, MnP particle sizes that have been found to cause harm (predominantly in controlled laboratory studies at non-environmentally relevant concentrations) are usually different (smaller) than MnP size ranges usually targeted in current analyses of samples recovered from soil, water, food, air, or the built environment. Nanoplastics in particular are inherently difficult to analyze in, or extract from, environmental matrices, resulting in a severely limited database on nanoplastic exposure. Microplastic particles are generally comfortably analyzed down to 10–20 μm sizes (by Fourier-transform infrared spectroscopy/Raman spectroscopy/fluorescence microscopy). However, analysis of sub-10 μm particles, the range considered of most concern for human health, is more challenging and resource intensive and therefore has not been widely considered in environmental exposure studies to date.

## Potential MnP influences on NCD prevalence and severity

**Figure 3:**
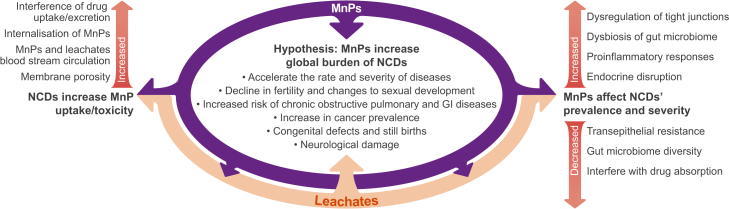
Hypothesized reciprocal interactions between MnPs in the human body and NCDs indicating the potential mechanisms of increased NCD prevalence and severity through MnP exposure as well as NCD linked increases in susceptibility to MnP uptake and thus toxicity This conceptual model synthesizes the links between the main exposure routes presented in Figure 1 (inhalation, ingestion, and dermal) and the impacts of MnPs including the systems and organs most affected by MnPs (respiratory system, gastrointestinal tract, cardiovascular system) as shown in Figure 2.

MnP exposure can cause physiological responses, such as chronic low-level inflammation, that are similar to the symptoms of many NCDs.^26^^,^^96^^,^^97^ We hypothesize that this may lead to the enhanced prevalence and severity of these NCDs.^98^ Initial evidence supports the hypothesis that inflammatory responses to MnPs may exacerbate or trigger flareups of existing NCDs related to the GI tract^98^^,^^99^ and cause potential cardiac toxicity, inducing problems such as hemolysis, thrombosis, blood coagulation, and vascular endothelial damage (various organisms, including human; Figure 3).^92^^,^^96^ In the GI tract, nuclear factor κB (NF-κB)-induced inflammation has been observed in response to polystyrene MnP exposure (zebrafish),^100^ causing effects similar to inflammatory NCDs such as Crohn’s disease and ulcerative colitis.^101^ MnP exposure has also been found to induce pro-inflammatory responses such as increased transcription of cytokine genes (zebrafish and human cell lines)^100^^,^^102^ and increased expression of immunomodulating agents, including interleukin (IL)-1α (rodent),^102^ IL-1β, IL-8, and NF-κB (zebrafish).^100^ MnP exposure to human liver resulted in hepato-, lipo-, and cytotoxicity, specifically causing increased expression of hepatic HNF4A and CYP2E1, which has been linked to increased risk of liver steatosis, fibrosis, and cancer.^103^ Furthermore, MnPs have shown potential to cause dysbiosis within the GI microbiome of rodents, resulting in inflammation and oxidative stress^25^^,^^26^^,^^104^ and further damage to the already impaired antioxidant-rich mucosa in inflammatory bowel conditions.^105^^,^^106^ Oxidative stress, inflammation, and interactions of MnPs and cellular components are also highlighted as the main mechanisms for cardiovascular toxicity.^96^^,^^97^ Further evidence exists with regards to the dysregulation of tight junctions that mediate the permeability of the GI epithelial membrane, a symptom of ulcerative colitis and Crohn’s disease.^23^^,^^107^ Such dysregulation can be triggered by the inflammatory mechanisms induced by MnP exposure in the GI tract *in vitro*, such as expression of NF-κB, tumor necrosis factor (TNF)-α, and IL-1, leading to increased GI permeability.^102^^,^^108^ Indeed, polystyrene MnPs increased the tight junction permeability of Caco-2 cell monolayers, although biological and chemical transformations of the MnPs during the digestive process mitigated this effect and increased the levels of pro-inflammatory cytokines.^102^

As evidenced by initial cellular and animal experiments, exposure to MnPs may lead to toxicological impacts on cell metabolism and cell-cell interactions,^90^^,^^109^ effecting the digestive, respiratory, endocrine, reproductive, and immune systems. Detailed mechanistic investigations of respiratory impacts caused by MnP exposure are still sparse, although evidence is emerging to support the hypothesis that inhalation of MnPs can affect respiratory NCDs (Figures 2B and 2C). Despite the existing knowledge gaps, the use of the adverse outcome pathway (AOP) framework and knowledge base (AOPwiki) is helping to connect the dots from particle (including MnP) exposure to the induction of adverse effects via oxidative stress and inflammatory responses. For instance, AOP173 suggests that exposure to persistent particles (such as MnPs) may trigger lung fibrosis, a dysregulated or exaggerated tissue repair process denoted by the presence of scar tissue in the localized alveolar capillary region of the lung where gas exchange occurs, which occurs as a result of non-resolving inflammation and ensuing tissue injury.^110^ A draft AOP linking MnP exposure through oxidative stress, inflammation, and apoptosis (cell death) to increased cancer (AOP505) was proposed by Jeong and Choi.^111^ Additionally, there is strong evidence of pollution from airborne combustion particulates affecting asthma, COPD, ischemic heart disease, atherosclerosis, cardiovascular disease, and cancer.^112^ At a cellular level, human lung epithelial cells exposed to polystyrene MnPs displayed cytotoxic and inflammatory effects, and decreased transepithelial electrical resistance indicative of tight junction dysregulation and potentially increased risk of COPD.^28^ Polystyrene MnPs internalized by human lung epithelial cells (BEAS-2B) can cause autophagic reticulum stress-related metabolic changes resulting in cell dysregulation and decreased resistance to cytotoxic effects.^113^ Polystyrene particles are internalized by human (A529) epithelial cells, resulting in significant up-regulation of pro-inflammatory cytokines (IL-8, NF-κB, TNF-α), activated inflammatory gene transcription, and protein expression.^114^ When “natural” inhalation rather than direct cell exposure is assessed, rat lung epithelial cells illustrate an exposure-concentration-dependent inflammatory protein expression (TNF-α-and TGF-β),^115^ complementing previous cellular studies and demonstrating the inflammatory response of lung epithelial cells to MnP exposure.

## The consequences of increased systemic circulation of MnPs

We propose that the effects of inhaled and ingested MnPs are not limited to the initial exposure sites but that MnPs can penetrate the circulatory system, potentially as a result of tight junction dysregulation (Figures 2B–2D) affecting even distal organs. MnPs smaller than 2.5 μm can impair the epithelial membrane (Figure 2C), resulting in translocation of MnPs into the bloodstream and potentially throughout the body.^116^ For example, polystyrene MnPs have been shown to cause increased inflammation and possible neurological changes in rodents.^115^^,^^117^ MnPs can be actively transported across mucosal membranes into the circulatory system by adsorption of specific proteins, allowing them to interact with endocytic receptors^102^^,^^118^ or even to transfer across an *ex vivo* placental blood barrier.^118^ In this way, MnPs may create a Trojan horse effect whereby environmental contaminants such as persistent organic pollutants, heavy metals, and bacteria may be adsorbed to particle surfaces and thus be transported into the body concurrently.^27^^,^^119^ Recent research also found MnPs in the heart, spleen, placenta, and fetus of rodents^96^^,^^120^ as well as in the human placenta,^14^^,^^15^ meconium, infant feces, and baby milk (both breastmilk and formula),^11^^,^^13^ suggesting that it will be crucial to establish if MnP-induced inflammatory responses could trigger adverse outcomes in pregnancy. Indeed, preeclampsia and hypertension (cardiovascular inflammatory processes) are common causes of maternal and fetal morbidity and mortality,^121^ and it remains to be determined if these could be exacerbated by exposure to MnPs.

### Health risks associated with MnP leachates

In addition to the potential health effects related to MnPs acting as fine particles, there is risk of them causing negative health impacts through chemical pathways. Besides their actual polymers, MnPs are comprised of a heterogeneous mix of chemicals (e.g., polybromide diphenyl esters, phthalates, nonylphenols, bisphenols, antioxidants) and often act as passive collectors of contaminants from their surrounding environment.^12^ Both the “ingredient” chemicals and plastic additives as well as contaminants passively collected throughout their environmental fate may be released during MnP organismal uptake or degradation, causing risk of localized or distal health effects as they are circulated in the organism.^12^^,^^122^ The health effects of many of these compounds (e.g., bisphenols and phthalates), independent of their potential source being MnPs or not, are well established in the scientific literature (Figure 3), and their ubiquitous presence in both pediatric and adult populations^123^^,^^124^ is well known to cause human health issues.^125^^,^^126^ For many of these co-contaminants, it remains yet to be established how important MnP-associated contributions are as compared to other possible sources. Open questions remain regarding the capacity of smaller particles to pass the epithelial barrier where they are more likely to be contained in organs for longer and hence have a greater opportunity to leach toxic compounds. Similarly, questions remain to what degree MnP particle aging may lead to a reduction in leachate load before particles are taken up into organisms, as initial evidence points toward lower concentrations in environmentally aged MnPs than those found in pristine virgin particles.^127^

Simulated intestinal fluids have been shown to leach additives from MnPs into the local gut environment,^128^^,^^129^ causing increased transcellular permeability as evidenced by the detection of microbiome markers in blood serum and epithelial intracellular enzymes in feces.^104^ It is not yet known if any additive and/or synergistic adverse effects are associated with the presence of both MnPs and their compound-specific leachates. MnP leachates including endocrine-disrupting substances such as phthalates (e.g., di-(2-ethylhexyl) terephthalate in polyvinyl chloride) and other plastics or bisphenols including bisphenol A (BPA) can block the action of androgens, limit their biosynthesis, or promote estrogenic effects. These chemicals can cause cryptorchidism, hypospadias, decreased fertility, and increased susceptibility to certain cancers.^130^ Despite recent efforts to limit the use of BPA through legislation, it is commonly replaced by structural analogs such as bisphenol S and bisphenol F, which recent research has demonstrated to also elicit endocrine-disrupting responses.^131^ Polybrominated diphenyl ethers (PBDEs), a common organo-bromide chemical class used as flame retardants in plastic manufacturing, are widely applied to materials (e.g. seating foam and coverings, mattresses, and carpets), resulting in widespread human exposure.^132^ As also endocrine-disrupting chemicals they are affecting the hypothalamic-pituitary-thyroid axis disrupting the synthesis and transport of thyroid hormones impacting upon thyroid function.^133^^,^^134^^,^^135^ In addition, per- and polyfluoroalkyl substances (PFASs), a chemical class involved in the synthesis of plastics and PBDEs, have been found to alter telomere length and are linked to cancer (lengthened telomere sequence), cardiovascular disease, obesity, and premature death (shortened telomere sequence).^136^^,^^137^ Prenatal exposure to PBDE, PFAS, and polychlorinated biphenyls can cause an increase in IL-6 and TNF-α pro-inflammatory cytokines and a decrease in IL-10, resulting in increased inflammation during pregnancy and the postpartum period.^138^ Another common plasticizer, diethylhexyl phthalate, has been shown to increase the expression of the *MDR1* gene in the LS147T cell line, a model for colon carcinoma, which is suspected of inducing drug resistance to chemotherapeutic agents.^139^^,^^140^ This leads to an indirect impact of the body’s inflammatory mechanisms leading to sustained or increased inflammation, which we propose may lead to increased MnP uptake and further aggravation of NCDs. Investigations of plastic leachates such as BPA have shown the ability of leachates to cross the placenta and impact upon the neurological development of unborn offspring in mice.^141^^,^^142^ A growing body of evidence suggests that BPA and phthalates are capable of transplacental transfer to the unborn fetus while being present in the unmetabolized biologically active form.^143^ This transfer across the placenta has been associated with changes in DNA methylation and gene expression, which has unknown consequences for the fetus.^144^ There also appears to be a sex-specific and phthalate-exposure-related influence on birth weight and gestation,^145^ with suboptimal growth and preterm birth occurrence related to maternal phthalate exposure.^122^^,^^145^^,^^146^^,^^147^

### Potential reciprocity between NCDs and MnP uptake

In addition to MnPs potentially exacerbating NCDs by inducing additional inflammatory responses, there is the risk that pre-existing NCDs can increase MnP uptake, translocation, and impacts throughout the body (Figure 3). NCDs present significant inflammation of epithelial membranes, resulting in tight junction dysregulation and increased membrane porosity, potentially allowing greater uptake of MnPs via paracellular transport and subsequent translocation of MnPs throughout the cardiovascular system and to other organs, inducing inflammatory responses in organs that were previously unaffected by the respective NCDs (Figure 3). New findings forging into this research area have identified increased insulin resistance associated with 1 μm polystyrene MnPs ingested by mice as a result of colon and liver inflammation.^99^ While the role of NCDs in the uptake of MnPs is critically under-studied, we can build on our understanding of NCD-inflamed epithelial membranes, tight junction dysregulation, and increased permeability. In addition to MnP impacts at the initial exposure sites, it is also necessary to consider the increased MnP uptake and associated enhanced (distal) inflammatory responses when NCD conditions pre-exist.

## Recommendations: A one-health approach for characterizing MnP health risks

Addressing the knowledge gaps regarding the impacts of MnPs on human health outlined in this review will require the adoption of a one-health approach^148^ integrating transdisciplinary research including ecology, chemistry, engineering, biology, epidemiology, sociology, economy, and others. This includes a better understanding of the transport and fate of MnPs from environmental pollution sources to the human body while also understanding how health is impacted (microbiome, inflammatory reactions, enzyme modulation, drug resistance, etc.) by both particles and leachates. It is essential to improve the empirical evidence of MnPs dose-response effects and the transport mechanisms that control particle and leachate concentrations in human tissue, including particle size fractionation by the different biological barriers (Figure 2E) and its impacts on total MnP particle numbers, mass, and leaching potential. These pressing research gaps can be addressed by epidemiological studies linking a range of different exposure levels to potential outcomes (e.g., through matched case-control studies), tissue analysis studies to define exposure and link to disease outcomes, clinical studies to assess exposure and penetrance of MnPs into healthy vs. inflamed tissues, and mechanisms leading from MnP exposure to disease (adverse) outcomes.^97^^,^^149^ This, however, requires addressing the lack of appropriate standardized methods for quantifying and characterizing MnPs first, before useful epidemiological studies can even be conducted. While datasets from MnP human biomonitoring studies are still limited at present, similarities between MnPs and other particulate matter (e.g., anthropogenic air pollution particles) and engineered nanomaterials, for which more extensive evidence of particle biodistributions, biokinetics, and translocation across biological barriers exists,^150^ provide confidence that most plastic types have the potential for internalization following exposure via food or air.

After determining whether exposure to MnPs poses a health risk, it will be essential to establish the influence of socio-economic factors (diet, risk exposure, leisure activities, capacity to use alternative products, etc.). MnP uptake, leaching potential, and impact on NCDs are dependent on environmental exposures as well as biological and lifestyle factors (Figure 1). Systematic investigation of environmental and behavioral controls of MnP exposures linked to adverse health effects is required to fully establish the drivers of health-relevant uptake pathways. It will be crucial, therefore, to understand how other factors such as lifestyle (e.g., diet, dependence on bottled water as main source, smoking) and place of living may determine the severity of MnP dosage, while other biological factors (e.g., age, disease state) are likely to affect an individual’s susceptibility to MnP exposure. Exposure to MnPs can also be affected by regional variabilities (e.g., highly populated vs. remote areas, regional disease prevalence, and risk factors).

Making all these connections can be achieved through (1) improving the capacity for MnP and leachate detection in organisms and their environment, (2) mechanistically investigating MnP fate in organisms (including degradation and additive leaching), and (3) advancing functional studies of MnP impacts using realistic concentrations based on measured exposures. Epidemiological studies are also required to enable the modeling of exposure to MnPs and their contribution to the burden of diseases globally, in order to drive innovation in intervention development and large-scale action to improve health equity globally.

### Improving MnP environmental detection capacity to advance understanding of MnP exposure and fate in humans

Detection of MnPs in air, food, drink, and other pathways into the human body is limited by current analytical technologies as well as the lack of validated and standardized sampling, extraction protocols, and characterization methodologies. The capabilities of analytical techniques and their limits of detection are still being pushed with regard to MnP particle sizes and concentrations. Current studies of environmental exposure have analyzed MnP particles down to ∼2 μm in the air using μRaman spectroscopy,^30^^,^^151^ with one study providing concentrations for sub-200 nm particles using a thermal-desorption proton-transfer-reaction mass spectrometer, presenting results above the limit of detection of 10 ng/mL^38^ and down to 1 μm in drinking water (again using μRaman spectroscopy).^152^ However, the majority of studies so far have focused on the detection of MnP particles of 20 μm or larger using methods including pyrolysis-gas chromatography-mass spectrometry, Fourier transform infrared spectroscopy, μRaman spectroscopy, and fluorescence microscopy.^46^^,^^153^^,^^154^^,^^155^ It is recognized that controlled studies using environmentally relevant concentrations representing relevant particle size distributions are needed to determine the thresholds of acute and chronic exposure relevant to the variety of MnPs. Biological barriers provide size-specific limits for the uptake of particles into the bloodstream.

Current knowledge of environment-specific exposures is still fragmented and often does not extend to smaller MnP particle sizes in the nano range. State-of-the-art particle tracing technologies, such as the synthesis of MnPs doped with rare metals or tagged with fluorescent labels,^120^^,^^156^ provide the potential to alleviate existing detection limitations. These methods allow for quantifying uptake and localization of MnPs by inductively coupled plasma-mass spectrometry or fluorescent microscopy, respectively, to correlate the internalized dose with adverse effects. While the uptake of these methods is still limited (e.g., to tracking fate and effects in the environment, since ethical concerns would rightly limit intentional human exposure experiments), they are effective for modeling MnP behavior in lab-based environments. For example, they provide unprecedented insights into environmental aging mechanisms during organismal, tissue, and cellular uptake. In the future, it may be possible to apply such tagging techniques to nanoscale MnPs collected from the field. Current advances in new analytical methodologies such as thermal-desorption proton-transfer-reaction mass spectrometry,^38^ thermo-gravimetric analysis-Fourier transformed infrared, gas chromatography-mass spectrometry,^127^ double shot pyrolysis-gas chromatography-mass spectrometry,^155^^,^^157^ μRaman analysis (down to, and potentially below, 100 nm),^158^^,^^159^^,^^160^ and other emerging methodologies are promising to enable quantification and identification of environmental exposure, ingestion, and inhalation of MnPs <10 μm.

For quantifying direct and indirect effects of MnP exposure, it will be crucial to determine the controls of MnP pollution source activation and how environmental fate and transport pathways, including environment-specific degradation mechanisms, create connectivity between pollution sources and impacted organs and systems. It will therefore be essential to also advance the understanding of the rates of transport of MnPs and their leachates across different biological barriers as well as the drivers of MnP degradation and additive leaching in different biological systems before, during, and after uptake into humans (e.g., in the acidic gut or in acidic lysosomal compartments following endocytotic uptake). It is worthwhile for this to explore the transferability of concepts derived from, for instance, the transport of metallic nanoparticles or their ionic form across the blood-brain barrier,^161^ the transport of nanoparticles across the placental barrier using *ex vivo* placenta models,^162^^,^^16^ or investigations of the bidirectional transport of polystyrene MnPs across a human *ex vivo* placenta.^163^ Testing the applicability of such models to MnPs of different types, characteristics, and exposure scenarios is crucial for understanding human transmembrane transport of MnPs to better shape our understanding of MnP distribution and toxicity.

### Advancing functional studies of MnP impacts

Developing functional studies of MnP interactions with mucosal membranes, for instance by using organ-on-a-chip technology,^100^ spheroid cell cultures,^164^ or 3D cell cultures resembling human skin,^165^ provides an opportunity for cellular responses to MnP exposure to be characterized and correlated with specific MnP properties such as size, shape, composition, or additive compositions. Histological studies of mucosa and epithelium can elucidate inflammatory conditions, in conjunction with multiomic pathway analysis of cell-based models to further define the mechanisms of MnP toxicity. In this respect, the advancement of existing particle-based pharmacokinetic models to include MnPs will offer new ways to gain insights into the biodistribution, residency, and toxicity of MnPs.^166^ Utilizing technological advances such as animal disease models can help to determine synergistic or additive effects of MnP-induced inflammation in the presence or absence of underlying NCDs.

In addition, there is potential to leverage relevant transferable knowledge from nanosafety research and air pollution health effect studies using ultrafine particles and PM_10_/PM_2.5_ that can provide important mechanistic understanding and help to close existing knowledge gaps in the understanding of the functional consequences of MnP uptake for NCD induction, severity, and susceptibility.

## Conclusion: Global health implications

Given increased awareness of the global reach of plastic pollution and the persisting gaps in understanding the potential impacts of these complex pollutants, concerted efforts toward developing a better assessment of the overall health risks associated with MnP exposure are critically needed. This becomes particularly important given the current limitations of remediation options. It is now widely acknowledged that MnP pollution needs to be addressed at the source, thereby preventing further emissions, as clean-up and remediation options are limited, and the global dispersion of MnPs that has already happened will remain a cause of concern for centuries to come even if we could “close the tap” of MnPs escaping into the environment right now.

Based on our review, we identified persisting knowledge gaps and propose a strategy for a systematic investigation of MnP impacts on NCD prevalence and severity that is urgently required to progress global efforts toward the UN Sustainable Development Goal (https://sdgs.un.org/goals) Target 3.4: to reduce premature mortality from NCDs through prevention and treatment by 2030. This need is particularly critical in low- and low-middle-income countries where NCD prevalence is rising and plastic pollution levels and exposures are high. MnPs are adding to the health risks arising from general particulate exposure and critically extend those exposures and risks into indoor spaces.

The existing evidence summarized in our review suggests that there is more than a hypothetical relationship between MnPs and NCDs, which will be crucial to unravel for assessing current and future health risks. There is rising awareness of the links between NCDs and pollution^167^^,^^168^ and increasing evidence that natural (e.g., pollen), anthropogenic (e.g., diesel exhaust, MnPs), and engineered nanomaterials all act in a similar biological manner and, by being treated as foreign entities by the body, can trigger the same protective mechanisms. Consequently, there is a real risk of these protective systems becoming overwhelmed, leading to “overload” conditions and resulting pathologies. While our hypothesis is built on evidence suggesting links between MnP exposure and NCDs, it is likely to apply similarly to infectious diseases that elicit inflammatory responses, and explicit links with other diseases need to be urgently explored. Leslie et al.^10^ also hypothesized that MnP particles present in the bloodstream that are being carried by immune cells could affect immune regulation or the predisposition to diseases with an immunological base.

Systematic assessment of human health risks and societal and economic burdens associated with MnP pollution will enable a more holistic assessment and leverage the design and implementation of integrated technological, regulatory, and behavioral solutions for risk reduction. By investigating these relationships further, we will be able to understand how they influence exposure and susceptibility and design meaningful recommendations to reduce the health risk associated with MnPs beyond the direct intakes (i.e., from inhalation and food). Therefore, we advocate for adopting a one-health approach that builds on increased collaboration between all scientists, but especially between health and environmental sciences, to develop strategies toward understanding and to ease the global health burden from increasing MnP exposures. Such a holistic approach will allow for a better understanding and prediction of fate and transport processes that affect MnP exposure, including the links between human behavior and activities impacting the pathways and uptake through food and agriculture.
